# BRCA1 gene polymorphism and finger dermatoglyphic patterns in Ghanaian breast cancer patients: a quantitative cross-sectional approach

**DOI:** 10.11604/pamj.2022.43.209.33136

**Published:** 2022-12-28

**Authors:** Emmanuel Osei Nkansah, John Ahenkorah, Kevin Adutwum-Ofosu, Raymond Lovelace Adjei, Nii Armah Adu-Aryee, Emmanuel Ayitey Tagoe, Nii Koney-Kwaku Koney, Nii Ayite Aryee, Bismark Afedo Hottor, Richard Michael Blay, Joe-Nat Clegg-Lamptey, Benjamin Arko-Boham

**Affiliations:** 1 Department of Anatomy, University of Ghana Medical School, University of Ghana, Accra, Ghana,; 2 West African Center for Cell Biology of Infectious Pathogens, University of Ghana, Legon, Accra, Ghana,; 3 Department of Surgery, University of Ghana Medical School, University of Ghana, Accra, Ghana,; 4 Department of Surgery, Korle-Bu Teaching Hospital, Accra, Ghana,; 5 Department of Medical Laboratory Sciences, School of Biomedical and Allied Health Sciences, University of Ghana, Accra, Ghana,; 6 Department of Medical Biochemistry, University of Ghana Medical School, University of Ghana, Accra, Ghana

**Keywords:** Breast cancer, finger dermatoglyphics, BRCA1 gene, single nucleotide polymorphisms

## Abstract

**Introduction:**

breast cancer development is linked to mutant single nucleotide polymorphism of breast cancer type 1 (BRCA1) gene usually harboured within exon 11. It has also been linked to finger dermatoglyphics where certain patterns have been associated with breast cancer. This study suggests a possible relationship between finger dermatoglyphic patterns and single nucleotide polymorphism of BRCA1 gene.

**Methods:**

in a quantitative cross-sectional approach, finger dermatoglyphic patterns were obtained using the ink method from 70 female breast cancer patients and 70 age-matched apparently healthy females. Approximately 5 ml of venous blood was obtained from each participant from which DNA was extracted from the white blood cells collected after centrifugation. DNA was amplified and sequenced and the data aligned with the wildtype template of BRCA1 gene. Fingerprint patterns were analyzed with Chi-square. Mean frequency of fingerprint patterns was analyzed with independent student’s t-test. Differences in data set with p<0.05 were statistically significant.

**Results:**

luminal B was the predominant breast cancer molecular subtype among the patients. The predominant fingerprint pattern among breast cancer participants was the loop. Six or more loops had higher frequency among breast cancer females. The predominant BRCA1 gene variant locations were c.34311, c.34320, and c.34321 with c.34311A>C being the predominant variant. Higher percentage frequency of six or more loops in relation to c.34311A>C was observed in apparently healthy females compared to breast cancer females.

**Conclusion:**

the study reports for the very first time in Ghana, BRCA1 gene variants and finger dermatoglyphics among breast cancer patients. Although the results are preliminary and inconclusive it creates an avenue for extended studies.

## Introduction

Cancer of the breast is a malignant disease that results from abnormal proliferation of cells within the breast tissue, which in advanced cases metastasize to other organs including bony skeleton, brain, lung and the liver [1,2]. Globally in 2020 alone, breast cancer affected 2.3 million women with 685,000 deaths. This contributed to the estimated 7.8 million women alive who had been diagnosed with breast cancer in the preceding 5 years (2015-2020), making the disease the world´s commonest malignancy [3]. In Africa, there is an increasing incidence of the disease with relatively high mortality due to late diagnosis and treatment. Breast cancer incidence in Ghana in 2020 was 4,482 out of which 2,055 died, a mortality rate of nearly 46% [4]. Breast cancer, a heterogeneous disease, is representative of numerous subcategories of several cellular compositions, molecular alterations and clinical behaviour. The molecular subtypes of breast cancer are specified based on statuses of the oestrogen and progesterone receptors, human epidermal growth factor receptor 2 (HER2) and Ki-67 within the luminal and myoepithelial cells. They include; luminal A, luminal B, triple-negative, HER 2 enriched and normal-like breast cancers [5,6].

It has been established that breast cancer development has genetic undertones with breast cancer susceptibility genes 1 and 2 (BRCA1 and BRCA2) as the most famous [7]. Breast cancer type 1 gene is a tumour suppressor gene located on chromosome 17q21 and has DNA repair ability [8]. Mutations in the gene has been associated with breast cancer development with reports suggesting that about 72% of women inheriting a mutated BRCA1 gene develop breast cancer by the age of 80 years [7,9]. A common source of genetic mutation is single nucleotide polymorphisms (SNPs). Single nucleotide polymorphisms are genetic variations that occur at a specific locus within genomic sequence where a single nucleotide adenine (A), thymine (T), cytosine (C) or guanine (G) is altered [10,11]. Single nucleotide polymorphisms of BRCA1 gene, therefore, result from a change of a single nucleotide within the genomic sequence of BRCA1 gene in a human population and presents as a homozygous wild, heterozygous carrier or a homozygous mutant. The mutant form of the SNPs of BRCA1 gene has been reported to be associated with the risk of breast cancer development [12,13].

Finger dermatoglyphics is the scientific study of the skin ridge patterns on the palmar surface of the distal end of the fingers [14]. Three (3) broad types of fingerprints patterns have generally been described: loop, whorl and arch [15,16]. Fingerprint pattern formation is intrauterine and genetically influenced, and remains stable and unique for an individual throughout life. As a result, finger dermatoglyphics have gained tremendous forensic utility and for many decades have been explored for their usefulness in screening for genetic diseases, including familial breast cancers [17,18]. While Chintamani *et al*. [17] reported that six or more whorls were significantly higher among females with breast cancer compared to females without breast cancer, Natekar and colleagues [18] observed six or more loops to be significantly higher among breast cancer females compared to healthy females. These reports among many similar others, give inspiration to consider finger dermatoglyphics as potential non-invasive anatomical marker for screening diseases including the assessment of risk of breast cancer development. The present study aimed at and reports on the relationship between the single nucleotide polymorphism of BRCA1 gene and finger dermatoglyphic patterns in breast cancer patients. The specific objectives included: (1) to determine the predominant location of single nucleotide polymorphism and the resulting nucleotide variants of BRCA1 gene (exon 11 and surrounding introns) among study participants; (2) determine the predominant finger dermatoglyphic patterns in individuals with breast cancer and individuals without breast cancer; (3) to ascertain tumour characteristics in individuals with breast cancer in relation to fingerprint patterns and; (4) to assess the predominant variants of BRCA1 gene in relation to the predominant finger dermatoglyphic pattern among individuals with breast cancer and individuals without breast cancer. We hypothesized that there was no relationship between finger dermatoglyphic patterns and BRCA1 gene polymorphism among individuals with breast cancer.

## Methods

**Study site, study design, and participant recruitment:** a quantitative cross-sectional design was adopted for the study. Seventy (70) females clinically diagnosed with breast cancer and undergoing chemotherapy at the Oncology Unit of the Department of Surgery, Korle-Bu Teaching Hospital were recruited through simple random sampling. Additionally, seventy (70) age-matched apparently healthy females within the immediate environment of the Korle-Bu Teaching Hospital who met the inclusion criteria were recruited through simple random sampling. Control patients provided responses through interviews to exclude those with self-reported clinical conditions such as hypertension, diabetes, and cancers. Clinical and histopathological information including molecular sub-types on breast cancer patients were obtained from the hospital folders of each consented breast cancer participant. The sample size was estimated using the formula


$$N=\frac{2{\left(Z_{\alpha +Z\beta }\right)}^{2}\sigma }{\Delta^{2}}$$

α predicts the probability of making a Type I error and β predicts the probability of making a Type II error, where α is the significance level, α for this study = 0.05. Z_α_= A standardized normal deviation value that corresponds to a level of statistical significance (0.05) equals 1.96, Z_α_=1.96. β = power, probability of detecting a significant result (typically 80%, 90%). β for this study was 80%=0.20. Z_β_= probability of detection or power (80%), Z_β_=0.84. β is the standard deviation for the data (in similar studies, σ = 1). Δ = the effect size. Effect sizes as small, moderate, and large (0.2, 0.5, and 0.8 for two-group comparisons. Solving for N resulted in N= 63. The total number of breast cancer participants was rounded to seventy (70) with an equal number of apparently healthy participants.

**Fingerprint pattern acquisition:** finger dermatoglyphic patterns were obtained using the ink method. Each participant was directed appropriately to dip the palmar surface of the distal part of the fingers of each of the ten (10) fingers in dry ink. The ten (10) inked fingers of each participant were then pressed on a piece of white paper, one finger at a time. The resulting fingerprints were identified and recorded.

**Blood sampling and preparation:** by venipuncture using the median cubital vein, a total of 5 milliliters of peripheral blood was collected from each study participant into Ethylenediaminetetraacetic acid (EDTA) coated test tube. Samples were centrifuged at 3000rpm for 5 minutes to separate the blood components. The plasma was gently aspirated into an Eppendorf tube to make the buffy coat accessible. The buffy coat (containing the white blood cells) was then aspirated into fresh labeled Eppendorf tubes using a Pasteur pipette and placed in a cryo-box before storage at -20°C until use.

**Deoxyribonucleic acid (DNA) extraction:** DNA was extracted from the white blood cells within the buffy coat using the Zymo Research Kit [Zymo Research, USA]. Five hundred (500) microliters of genomic lysis buffer [Inqaba Biotechnical Industries (PTY) Limited, South Africa] was added to 150 μl of thawed buffy coat. The mixture was vortexed for 5 seconds and then let to stand for 10 minutes at room temperature. The mixture was transferred to a zymo-spin column placed in a collection tube and centrifuged at 10,000 xg for 1 minute. The collection tube together with the flow-through was discarded. The zymo-spin column was transferred to a new collection tube. Subsequently, 200 μl of DNA pre-wash buffer was added to the spin column and centrifuged at 10,000 xg for one minute. Five hundred (500) microliters of gDNA wash buffer were then added to the spin column and centrifuged at 10,000 xg for 1 minute. The spin column was then transferred to a clean microcentrifuge tube. 40 μl of DNA elution buffer was added to the spin column and incubated for 5 minutes and then centrifuged at 10,000 xg for 30 seconds to elute the DNA. The purity and concentration of the eluted DNA were determined using a NanoDrop Spectrophotometer. The purity of 1.8-2.0 (A260/A280) and concentration of ≥20 ng/μl were required to qualify the eluted DNA as good. The extracted DNA served as a template DNA for the polymerase chain reaction.

**Polymerase chain reaction (PCR) procedure and conditions:** polymerase chain reaction (PCR) was used for amplifying the DNA extracted using appropriate primers. A total reaction mixture of 25 μl was constituted for cycling in each reaction tube. It was composed of master mix (12.5 μl), nuclease-free water (10.5 μl), template DNA (1 μl), BRCA1 forward primer (0.5 μl) and BRCA1 reverse primer (0.5 μl). The reaction mixture was placed in a thermocycler (Prime thermal cycler, Bibby Scientific Limited) and ran for 29 cycles with the following reaction conditions: initial denaturation at 94°C for 5 minutes; denaturation at 94°C for 1 minute, annealing at 62.5°C for 3 minutes, an extension of DNA strand at 72°C for 3 minutes and final extension at 72°C for 3 minutes. The PCR products were subsequently subjected to agarose gel electrophoresis to visualize and confirm correct amplification.

**Agarose gel electrophoresis:** agarose gel (1.5%) was prepared by weighing 1.5 g of agarose powder [Bio-Rad, USA] and mixed with 100 ml of Tris-acetate-EDTA (TAE) buffer. The 1.5% Agarose mixture was heated till boiling on a hot plate. The beaker was covered with aluminum foil to prevent evaporation of the mixture during heating. The mixture was then removed from the hot plate and allowed to cool after which 2 μl of ethidium bromide [New England Biolabs, USA] was added and then poured into a casting tray (with a comb fitted) and allowed to solidify. The electrophoresis tank was disinfected with 70% ethanol before the running buffer (1XTAE) was introduced. The solid gel which was within the casting tray was placed in the electrophoresis tank. Approximately 8 μl of PCR product from each sample was loaded into separate wells in the gel. A 100bp DNA ladder [Inqaba Biotechnical Industries (PTY) Limited, South Africa] was used as a molecular marker. Gel electrophoresis was run for 45 minutes at 100V. After gel electrophoresis, the gel with the amplicons was visualized using an Amersham imager (General Electric Healthcare Manufacturing Company, USA).

**Breast cancer type 1 gene sequencing:** having confirmed the successful amplification of the target gene, the aliquots of PCR products of samples were shipped to Inqaba Biotechnical Industries (PTY) Limited, South Africa for BRCA1 gene sequencing. The forward strands of the amplified DNA (BRCA1 gene) of samples specifically exon 11, with upstream and downstream introns were sequenced by Sanger Sequencing. The BRCA1 forward and reverse primers were, respectively, 5’CACACAGCTAGGACGTCATC-3’ and 5’TCCATCAAGGTGCTTACAGTC-3’.

**Single nucleotide polymorphisms determination:** when the sequencing results were received, Molecular Evolutionary Genetics Analysis software (MEGA X) was used to retrieve forward sequences of BRCA1 gene exon 11 and surrounding introns. Benchling® software was used to align the forward sequence of the BRCA1 gene (template or wild type) and the forward sequences of the amplified BRCA1 gene from study participants. After alignment of the forward sequences, nucleotides present on the amplified BRCA1 gene which mismatched the template sequence at specific locations were noted as SNPs.

**Statistical analysis:** data was entered and cleaned using IBM SPSS Statistics 20. Data on molecular subtypes among breast cancer and fingerprint patterns were summarized using tables and analyzed with Chi-square. The mean frequency of fingerprint patterns among study participants was analyzed with independent samples student t-test using Graph pad prism version 8 software. Six or more whorls and six or more loops were analyzed with Chi-square. The normality test confirms the normal distribution of data (p > 0.05) before conducting the independent samples t-test. Differences in data sets with p < 0.05 were considered statistically significant.

**Ethical considerations:** ethical approval for the study was obtained from the Ethics and Protocol Review Committee of the College of Health Sciences, the University of Ghana with Protocol Identification Number CHS-Et/M2-5.6/2019-2020. Participation in the study was voluntary and informed consent was sought before recruitment. Participants were given the liberty to withdraw from the study at any time with no consequences.

**Funding:** the Department of Anatomy of the University of Ghana Medical School, partly funded the research through its postgraduate research support fund.

## Results

**Age distribution among study participants:** breast cancer females were age-matched with apparently healthy females; therefore, the age distribution was similar for both groups. More than 50% of the participants were pre-menopausal. The age groups of 20-30, 31-40, 41-50, 51-60, 61-70, 71-80 and 81-90 years recorded, respectively, 1, 18, 19, 19, 8, 4 and 1. The minimum and maximum ages of study participants (breast cancer females and apparently healthy females) were 29 and 85 years, respectively. The modal age was 47 years. Generally, individuals 40 years and below were classified as “younger women” while those 41 years and above were classified as “older women”. Of the 70 breast cancer patients, 19 (27%) were younger and 51 (73%) older.

**Clinico-pathological characteristics of breast cancer participants:** in 50% of the breast cancer participants, the pathology affected the left breast, while in 47% of the cases, it was the right breast. In about 3% of the cases, the pathology was bilateral. In 11 of the 19 “younger” cancer patients, the left breast was affected while in 8 it was the right breast. The “older” group of 51, 24 and 25 individuals, respectively, had the left and right breasts affected and bilateral in 2 cases. Of the 70 breast cancer participants, 49 (70%) had been diagnosed with invasive carcinoma of no special type, 18.6% with invasive ductal carcinoma, and 4.3% with mixed carcinoma (mucinous carcinoma and invasive carcinoma of no special type). Other less frequent histopathological subtypes are shown in Table 1.

**Table 1:** histopathological diagnosis of breast cancer among participants

Histopathological diagnosis	Frequency (%)
Invasive carcinoma of no special type	49 (70.0)
Invasive ductal carcinoma	13 (18.6)
Mixed carcinoma (mucinous carcinoma and invasive carcinoma of no special type)	3 (4.3)
Multinodular carcinoma	1 (1.4)
Invasive cribriform carcinoma with extensive in situ component	1 (1.4)
Intracystic papillary carcinoma	1 (1.4)
Extensive high-grade ductal carcinoma in situ (DCIS) with stromal invasion and lymph node metastasis	1 (1.4)
Invasive lobular carcinoma	1 (1.4)

Of the breast cancer participants, 48.6% were diagnosed with Grade II, 35.7% with Grade III and 12.9% with Grade I (Table 2). Stage IIIB was the commonest (34.3%) followed by stage IIIA (21.4%) and stage IIB (14.3%). Six (8.6%) of the participants were at stage IV (Table 2). Concerning molecular subtypes, luminal B was predominant among the patients (Table 2). Twenty-five (25) out of the 70 breast cancer patients representing 35.7% were diagnosed with luminal B. The second most frequent subtype was the triple-negative subtype (25.7%) followed by HER2 enriched subtype (21.4%) and then Normal-like breast cancer 9 (12.9%). The least frequently diagnosed molecular subtype was luminal A. For two of the cases, information on the molecular subtype was not available.

**Table 2:** stage, grade and molecular subtype of breast cancer among study participants

Stage and grade of breast cancer	Frequency (%)
**Stage**	
IA	2 (2.9%)
IIA	5 (7.1%)
IIB	10 (14.3%)
IIIA	15 (21.4%)
IIIB	24 (34.3%)
IIIC	4 (5.7%)
IV	6 (8.6%)
Right breast-IIIB and left breast-IIIC	1 (1.4%)
Not available	3 (4.3%)
**Grade**	
I	9 (12.9%)
II	34 (48.6%)
III	25 (35.7%)
Not available	2 (2.9%)
**Molecular subtypes of breast cancer**	
Luminal B	25 (35.7)
Triple negative	18 (25.7)
HER2 enriched breast cancer	15 (21.4)
Normal-like breast cancer	9 (12.9)
Luminal A	1 (1.4)
Not available	2 (2.9)

**Fingerprint patterns among study participants:** generally, the loop was the predominant fingerprint pattern on all right fingers of breast cancer participants. In apparently healthy participants, the loop fingerprint pattern had the highest frequency on the right index finger, right middle finger, right ring finger and right little finger. However, there were more whorls than loops on the right thumb (Annex 1). The right ring fingers of breast cancer participants did not have an arch fingerprint pattern. On the left hand, the loop was the predominant fingerprint pattern among breast cancer participants. In apparently healthy participants, the loop fingerprint pattern had the highest frequency of occurrence on the left index fingers, left middle fingers, left ring fingers and the left little fingers, however, there were more whorls than loops on the left thumb (Annex 1). Among breast cancer participants, the mean frequencies of fingerprint patterns for loop, whorl and arch were 48.6, 14.9 and 6.5, respectively; and for the control participants, 42.2, 19.6 and 8.2 in the same order. There was, however, no significant difference between the two groups.

**Six or more loops and six or more whorls among study participants:** comparing the presence of six or more loops or whorls among breast cancer participants and controls, no difference was observed. Although six or more loops were more common among breast cancer participants, compared to apparently healthy participants, but the difference was not statistically significant (p = 0.61). Also, six or more whorls had higher frequency among apparently healthy participants compared with breast cancer participants, but the difference was not statistically significant, (p = 0.30). Table 3 summarizes the presence of six or more loops or whorls in relation to tumour characteristics in breast cancer patients. Out of 70 cancer participants, 51 recorded six or more loops. Of the 51, 19 (37.3%) diagnosed with luminal B had six or more loops. Regarding the tumour staging and grading, stage III and grade II individuals recorded the highest frequencies [32 (62.7%)] and [26 (51.0%)] respectively, of six or more loops. The association between the presence of six or more loops with tumour characteristics determined by Cramer´s V was very low. The highest Cramer´s V of 0.124 (p = 0.793) was observed between six or more loops and the breast cancer stage.

**Table 3:** finger dermatoglyphic patterns and tumor characteristics

Tumor characteristics	Frequency of presence of six or more loops (%); N = 51
**Molecular subtypes**	
Luminal A	7 (10.3%)
Luminal B	19 (27.9%)
Triple negative	13 (19.1%)
HER 2 enriched	10 (14.7%)
**Site of breast cancer**	
Left breast	25 (35.7%)
Right breast	25 (35.7%)
Bilateral	1 (1.4%)
**Stage**	
I	1 (1.5%)
II	12 (17.9%)
III	32 (47.8%)
IV	4 (6%)
**Grade**	
I	6 (8.8%)
II	26 (38.2%)
III	17 (255)

**Figure 1:**
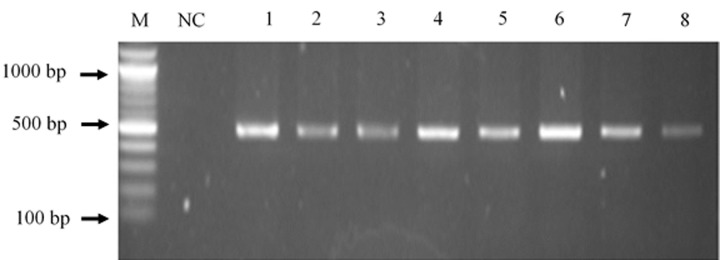
the image of a gel showing bands from amplicons after gel electrophoresis; M is 100bp molecular ladder; lanes 1,2,3,4,5,6,7 and 8 have bands showing amplification of BRCA1 gene of 450 base pairs; NC is the negative control

**Single nucleotide polymorphism of BRCA1 gene:** Figure 1 shows the average size of the bands representing the amplified BRCA1 gene as approximately 450 base pairs. After alignment of the forward sequence of the amplified BRCA1 gene to the forward sequence of the wild type of BRCA1 gene, a heterozygous allele, c.34311A>C had the highest frequency of occurrence in both breast cancer participants and apparently healthy participants (Annex 2). Variant location c.34320 was the second predominant location of nucleotide mismatch having two heterozygous alleles c.34320A>C (major) and c.34320A>T (minor) among study participants. The third predominant variant location was c.34321 and the resulting variant was c.34321A>T (Annex 2). These variants c.34311A>C, c.34320A>C (major) and c.34320A>T (minor), c.34321A>T are introns located upstream of exon 11.

**Molecular subtypes of breast cancer and variant location of BRCA1 gene:** out of the seventy (70) samples from breast cancer participants, 21 amplified DNA samples from breast cancer participants were selected through simple random sampling and sequenced. Out of the 21 breast cancer participants, 5 were diagnosed with luminal B breast cancer, 5 had triple-negative breast cancer, 5 had HER2 enriched breast cancer, 5 had normal-like breast cancer and one was diagnosed with luminal A breast cancer. From Annex 3, all 5 participants with HER2-enriched breast cancer had c.34311 as the predominant variant location, 4 participants with triple-negative breast cancer had c.34311 as the predominant variant location, 3 participants with normal-like breast cancer had c.34311 as the predominant variant location and 2 participants with luminal B breast cancer had c.34311 as the predominant variant location. Of the seventy (70) breast cancer participants only one was diagnosed with Luminal A and had the variant locations c.34311, c.34320 and c.34321.

**Fingerprint patterns (six or more loops) and predominant BRCA1 gene variants:** forty-eight per cent (48%) of breast cancer participants had six or more loops and c.34311A>C (Table 4). Also, 60% of apparently healthy participants had six or more loops and c.34311A>C. However, the difference in the frequency of occurrence of six or more loops and c.34311A>C among study participants was not statistically significant.

**Table 4:** fingerprint patterns (six or more loops) and predominant BRCA1 gene variants

Fingerprint patterns in relation to BRCA1 gene	Breast cancer participants; (N = 21)	Apparently healthy participants; (N =5)	P-value
Six or more loops and c.34311A>C	10 (48%)	3 (60%)	0.25
Six or more loops and c.34320A>T	3 (14%)	3 (60%)	<0.001*
Six or more loops and c.34321A>T	2 (9%)	3 (60%)	<0.001*

## Discussion

This study evaluated single nucleotide polymorphism of the BRCA1 gene and finger dermatoglyphic patterns in relation to tumour characteristics in breast cancer participants. BRCA1 mutations are responsible for approximately 40% of inherited breast cancers and more than 80% of families with both inherited breast and ovarian cancers. However, there has been limited study and therefore poor understanding of the role of BRCA1 in sporadic breast cancer. Several BRCA1 SNPs have been identified with some resulting in amino acid changes [7-13]. We report for the first time the existence of BRCA1 SNPs in Ghanaian females with breast cancer. The predominant variant locations observed are c.34311, c.34320, and c.34321 all resulting in introns located upstream of exon 11.

Exons are the coding sequences of a genome whereas introns are usually the non-coding sequences and are both involved in gene expression [19,20]. Single nucleotide polymorphism may occur in both exons and introns but alterations in the intronic sequences are more common than in exons [21]. Though the functional significance of the polymorphic intronic sequences is unknown, it is predicted that single nucleotide variants occurring within intron branch point sites, especially at the position with adenine (A), would presumably affect splicing [22].

Promoter regions are sites for the initiation of transcription of RNA and they are located upstream of exons and in eukaryotes usually contain TATA sequences which are bound by TATA-binding proteins to initiate transcription [23-25]. The three predominant variants c.34311A>C, c.34320A>C and c.34321A>T had adenine (A) replaced with cytosine (C), cytosine (C) and thymine (T), respectively. This would potentially disrupt BRCA1 transcription if any of the predominant variant locations were part of the promoter sites. Introns play a role in the regulation of gene expression and alternative splicing. Variations in intronic sequences may cause downregulation of gene expression and eventually lead to neoplasms [21]. However, c.34311A>C which was predominant in both breast cancer females and apparently healthy females indicates that the variant may be naturally occurring. Though we acknowledge the small sample size for this study as a limitation, this pilot study serves as the basis for further work to provide relevant information on the variations of introns and their association with neoplasms among Ghanaians.

In a participant with HER2-enriched breast cancer, however, a variant c.34474G>A was observed in exon 11 at c.34474 which resulted in an amino acid alteration; p. Arg1373Lys. The resulting amino acid, lysine is a basic amino acid just like arginine (the default amino acid) as such protein folding would not be adversely affected since lysine and arginine have similar qualities [26,27]. Exon 11 of the BRCA1 gene is the site for frequent mutations in breast cancer individuals and has been reported among breast cancer patients in Africa including populations from Nigeria, Egypt, Tunisia, Morocco, Algeria and South Africa [8,28,29]. Several studies have reported on the SNPs or mutations of the BRCA1 gene with regard to individuals with breast cancer in many countries [8,30-32]. Such information is unavailable in Ghana and to the best of our knowledge, this is the first report from Ghana setting the tone for further studies.

Of the different molecular breast cancer subtypes, triple-negative breast cancers reportedly common among younger women and African-Americans, are frequently characterized by BRCA1 mutations [6]. In this study, however, HER2-enriched breast cancer recorded the highest total frequency of BRCA1 mutations of 10 (Annex 3) followed by triple-negative. A subtype of HER2-enriched and luminal B breast cancers are noted for their fast growth and poorer prognosis [6,33] and their high prevalence among the study population may provide a window of explanation to why breast cancer among Africans and Ghanaians are thought to be generally more aggressive [34,35].

From Annex 3, the frequency of occurrence of the types of fingerprint patterns in reducing order was a loop, whorl and arch, an observation consistent with the literature [14]. Though fingerprint patterns are formed before the onset of diseases, they have over many decades been explored for their usefulness in diagnosis or predicting the risk of contracting certain diseases. We adopted and analyzed parameters such as six or more loops from Natekar *et al*. [18] and six or more whorls from Chintamani *et al*. [17]. Though six or more loops were higher among breast cancer participants compared to apparently healthy participants, the difference was not statistically significant since p > 0.05 (Annex 1). Also, six or more whorls had higher frequency among apparently healthy participants compared to breast cancer participants however, the difference was not statistically significant, since p > 0.05 (Table 4). Breast cancer participants besides having no arch on the right ring fingers had a higher frequency of six or more loops.

Apparently healthy participants had a higher percentage frequency of six or more loops in relation to c.34311A>C compared to breast cancer participants (Table 4) though the difference was statistically insignificant. Also, the higher percentage frequencies of six or more loops in relation to c.34320A>T and c.34321A>T among apparently healthy participants compared to breast cancer participants was noticed. We again emphasize that these observations may be affected by the small sample size of sequenced samples and thus, a larger sample size will be needed to validate our findings. Therefore, this information is insufficient to be used to explore the risk and prognosis of breast cancer, because six or more loops and the predominant BRCA1 gene variant, c.34311A>C were present in both breast cancer participants and apparently healthy participants and the difference in frequency of occurrence among study participants was not statistically significant.

In summary, we explored the molecular subtypes of breast cancer in relation to BRCA1 gene variants and fingerprint patterns. Key findings include: (1) there was the absence of arch fingerprint pattern on the right ring finger of breast cancer participants; (2) six or more loops had higher frequency among females with breast cancer compared to apparently healthy females; (3) c.34311, c.34320 and c.34321 are the predominant BRCA1 gene variant locations in the study population with c.34311A>C being the predominant variant; and (4) a higher percentage frequency of six or more loops in relation to c.34311A>C was observed in apparently healthy females compared to breast cancer females. The findings of this study, however, are preliminary and required extensive study to improve the generalizability. Limitations in the study include: (1) the small sample size of the sequenced BRCA1 gene from study participants potentially missing other relevant information and limiting the generalizability of study findings; and (2) inability to study details of fingerprint patterns such as subtypes of loops, whorls and arches as well as ridge counts, which could provide further information; and (3) inability to determine the duration of the disease prior to clinical diagnosis. This is solely because the clinical diagnosis was contingent on when patients reported to the hospital.

## Conclusion

This study sought to analyze the relationship between the single nucleotide polymorphism of BRCA1 gene and finger dermatoglyphic patterns in breast cancer patients and the potential usefulness of that information. We report that the predominant location in the studied population are c.34311 (c.34311A>C), c.34320 [c.34320A>C (major) and c.34320A>T (minor)] and c.34321 (c.34321A>T). The loop pattern was the commonest fingerprint pattern in both right and left hands in cancer patients similar to that exhibited by the controls. The study could not establish any tumour characteristic(s) that relate(s) to fingerprint patterns. The frequency of occurrence of six or more loops was common in c.34311A>C SNPs in both cases and controls. Besides the BRCA1 variants reported, the results are very preliminary and inconclusive and create an avenue for extended studies.

### What is known about this topic

- Breast cancer development is linked to mutant single nucleotide polymorphism of BRCA1 gene usually harboured within exon 11;
- Breast cancer has been linked to finger dermatoglyphics where certain patterns have been associated with breast cancer.

### What this study adds

- c.34311, c.34320 and c.34321 are the predominant BRCA1 gene variant locations in the study population with c.34311A>C being the predominant variant;
- Six or more loops had higher frequency among females with breast cancer compared to apparently healthy females.

## Annexes

**Annex 1**: fingerprint patterns of the right and left fingers of study participants- (PDF 422KB)

**Annex 2**: nucleotide variants and their location- (PDF 206KB)

**Annex 3**: molecular subtypes of breast cancer and variant location of BRCA1 gene- (PDF 423KB)
